# The complete chloroplast genome sequence of *Rhodiola sacra (Prain ex Hamet) S. H. Fu*

**DOI:** 10.1080/23802359.2019.1667275

**Published:** 2019-09-17

**Authors:** Kaihui Zhao, Yuanjiang Xu, Shiyuan Peng, Lianqiang Li, Hong Quan, Zhihua Liao, Xiaozhong Lan

**Affiliations:** a TAAHC-SWU Medicinal Plant Joint R&D Centre, Tibetan Collaborative Innovation Centre of Agricultural and Animal Husbandry Resources, Food Science College, Tibet Agriculture & Animal Husbandry University, Nyingchi of Tibet, China;; b Research Institute of Tibet Plateau Ecology, Tibet Agriculture & Animal Husbandry University, Nyingchi of Tibet, China;; c Key Laboratory of Eco-environments in Three Gorges Reservoir Region Ministry of Education), Chongqing Engineering and Technology Research Center for Sweetpotato, School of Life Sciences, Southwest University, Chongqing, China

**Keywords:** *Rhodiola sacra*, complete chloroplast genome, phylogenetic analysis

## Abstract

*Rhodiola sacra (Prain ex Hamet) S. H. Fu* is a traditional natural plant pharmaceutical with anti-hypoxia effect and mainly distributed in Yunnan and Tibet (China). The complete chloroplast sequence of *R. sacra* was determined in our study. The cpDNA was 150,941 bp in length, containing a pair of inverted repeats (IRs) of 25,873 bp each separated by a large and small single copy (LSC and SSC) regions of 82,161 bp and 17,034 bp, respectively. The genome contained 84 protein coding genes, eight rRNA genes and 36 tRNA genes. Phylogenetic tree revealed that *R. sacra* closely related to *Rhodiola kirilowii* and *Rhodiola crenulata*.

*Rhodiola sacra (Prain ex Hamet) S. H. Fu* is a drug source with anti-hypoxia effect, which is mainly used to strengthen the body and relieve the altitude sickness or discomfort caused by fatigue, belongs to the *Rhodiola L* genus, Crassulaceae family. The dry roots and rhizomes of the drug are medicinal part and used to nourish and clear the lungs (Hu et al. 2004). *Rhodiola L.* is an important and precious natural resource for new functional foods and medicines. *R. sacra* is one of the main medicinal plants of *Rhodiola L.* In this study, we assembled and characterized the whole chloroplast genome of *R. sacra* and learned more about genetic information of this species, which can contribute to the conservation, and provide useful help for population genetics studies of *R. sacra*. The annotated genome sequence has been deposited into Genbank under the accession number MN109978.

The plant materials of *R. sacra* were collected from Nyingchi (Tibet, China; N:29°32′20.56″, E:092°40′51.57″). The specimens of *R. sacra* have been kept in Tibet Agriculture & Animal Husbandry University and specimen Accession number is 542229180618172LY. Total genomic DNA of *R. sacra* was extracted from silica-gel-dried leaves using the modified CTAB method (Allen et al. 2006). Sequencing was carried out on the Illumina HiSeq 2000 platform (Illumina, San Diego, CA). A total of 2.0 Gb raw reads were obtained and then de novo assembled using CLC genome assembler program (ver.4.06 beta, CLC Inc, Aarhus, Denmark) as previously described (Kim et al. 2015). We assembled the chloroplast genome using Geneious Prime 2019.2.1 (Kearse et al. 2012), with *Sedum oryzifolium* (GenBank: NC027837) as the reference. DOGMA (Dual Organellar GenoMe Annotator) online program was used for annotation of the complete chloroplast genome (Wyman et al. 2004), and the annotation was corrected with Geneious Prime 2019.2.1 (Kearse et al. 2012).

The complete chloroplast genome of *R. sacra* (GenBank accession MN109978) was 150,941 bp in length with 37.80% GC content, consisting of a large single-copy (LSC) region of 82,161 bp and a small single-copy (SSC) region of 17,034 bp, separated by a pair of 25,873 bp IR region. A total of 128 genes were successfully annotated, containing 84 protein coding genes, 8 rRNA genes, and 36 tRNA genes.

The complete chloroplast genome sequences of *R. sacra* and 16 other species were used for phylogenetic analysis. The evolutionary history (Figure 1) was generated by maximum likelihood (ML) method of MEGA 7 (Kumar et al. 2016) using 1000 bootstrap replicates from alignments created by the MAFFT (Katoh and Standley 2013). We hope the complete chloroplast of *R. sacra* could provide a valuable resource for further evolution and phylogenomic studies in the genus and family.

**Figure 1.**
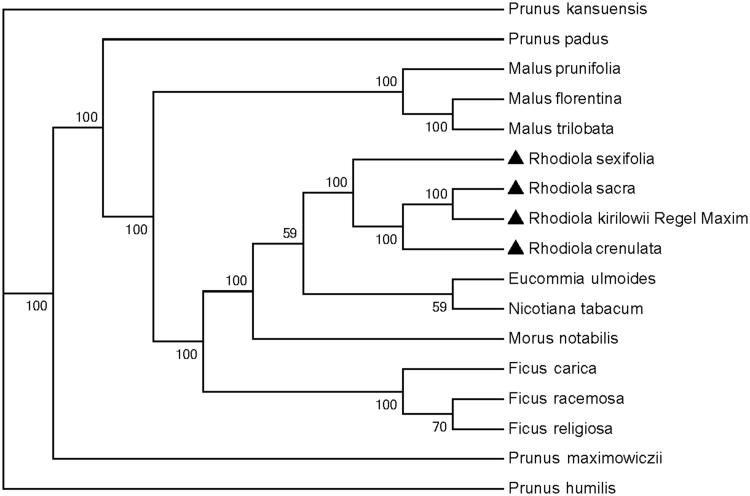
Phylogenetic tree based on the complete chloroplast genome sequences of *R. sacra* and 16 other species. The tree was generated using a ML method by MEGA7 with 1000 bootstrap replicates. Numbers on the nodes indicate bootstrap values. The chloroplast genome sequences used to construct the phylogenetic tree are MF766010, KY635880, KT368151, KY416513, KX499856, KU851961, KX499858, MK301435, MF405921, KF990036, KP760071, KP760072, Z00044, MN218690, MN109979, MN109978, MN109980.
